# The Clinical Resource Hub Telehealth Program and Use of Primary Care, Emergency, and Inpatient Care During the COVID-19 Pandemic

**DOI:** 10.1007/s11606-023-08476-x

**Published:** 2024-01-22

**Authors:** Kritee Gujral, Jennifer Y. Scott, Clara E. Dismuke-Greer, Hao Jiang, Emily Wong, Jean Yoon

**Affiliations:** 1 Health Economics Resource Center, VA Palo Alto Health Care System, Menlo Park, CA USA; 2 Center to Improve Veteran Involvement in Care, VA Portland Health Care System, Portland, OR USA; 3 Department of General Internal Medicine, UCSF School of Medicine, San Francisco, CA USA

## Abstract

**Background:**

The COVID-19 pandemic disrupted delivery of health care services worldwide. We examined the impact of the pandemic on clinics participating in the Veterans Affairs (VA) Clinical Resource Hub (CRH) program, rolled out nationally in October 2019, to improve access to care at under-resourced VA clinics or “spoke” sites through telehealth services delivered by regional “hub” sites.

**Objective:**

To assess whether the CRH program was associated with increased access to primary care, we compared use of primary, emergency, and inpatient care at sites that adopted CRH for primary care (CRH-PC) with sites that did not adopt CRH-PC, pre-post pandemic onset.

**Design:**

Difference-in-difference and event study analyses, adjusting for site characteristics.

**Study Cohort:**

A total of 1050 sites (254 CRH-PC sites; 796 comparison sites), fiscal years (FY) 2019–2021.

**Intervention:**

CRH Program for Primary Care.

**Main Measures:**

Quarterly number of VA visits per site for primary care (across all and by modality, in-person, video, and phone), emergency care, and inpatient care.

**Results:**

In adjusted analyses, CRH-PC sites, compared with non-CRH-PC sites, had on average 221 additional primary care visits (a volume increase of 3.4% compared to pre-pandemic). By *modality*, CRH-PC sites had 643 fewer in-person visits post-pandemic (− 14.4%) but 723 and 128 more phone and video visits (+ 39.9% and + 159.5%), respectively. CRH-PC sites, compared with non-CRH-PC sites, had fewer VA ED visits (− 4.2%) and hospital stays (− 5.1%) in VA medical centers. Examining visits per patient, we found that CRH-PC sites had 48 additional telephone primary care visits per 1000 primary care patients (an increase of 9.8%), compared to non-program sites.

**Conclusions:**

VA’s pre-pandemic rollout of a new primary care telehealth program intended to improve access facilitated primary care visits during the pandemic, a period fraught with care disruptions, and limited in-person health care delivery, indicating the potential for the program to offer health system resilience.

**Supplementary Information:**

The online version contains supplementary material available at 10.1007/s11606-023-08476-x.

## INTRODUCTION

The onset of the COVID-19 pandemic disrupted health care services globally.^1, 2^ Health systems began shutting down non-essential patient services in March 2020 to prevent the spread of COVID-19 infection. The disruption to care delivery posed threats of severely worsening health care access in already fragile rural and underserved areas that face perennial shortages of health care professionals. In response to the pandemic, health systems began making unprecedented shifts to delivering care remotely via telehealth modalities.^3^ Telehealth is broadly defined as “the use of electronic information and telecommunication technologies to support long-distance clinical healthcare.”^3, 4^ The pandemic emphasized the importance of telehealth, specifically synchronous audio-only and audio–video episodes of care between patients and clinicians,^5^ as a key strategy for maintaining and facilitating access to care during crisis times, with the potential to enable health system resilience.^1, 6–12^

However, very few large-scale or national studies have evaluated whether existing telehealth infrastructure facilitates delivery of health care during crisis times.^8, 13^ The lack of studies on this topic may, in part, be attributed to the historically low uptake of telehealth by health systems prior to the COVID-19 pandemic. The U.S. Veterans’ Health Administration (VA) is an important exception as it was a leading provider of telehealth services in the USA even prior to the COVID-19 pandemic.^14^ As such, VA programs and data allow opportunities to examine how existing telehealth infrastructure can help maintain access to care and minimize care disruptions if crises, such as the COVID-19 pandemic, arise.

In this study, we leverage VA’s existing telehealth infrastructure and a relatively more established culture of telehealth encounters between patients and providers to examine the provision of primary care facilitated by the pre-pandemic rollout of VA’s Clinical Resource Hub (CRH) telehealth program. Building on pilot programs, VA began implementing a national contingency staffing program in October 2019, ^11, 15–17^ just before the onset of the COVID-19 pandemic. CRH aimed to provide staffing on a contingency basis in cases of non-catastrophic staff deficits or gaps in health care supply at primary and mental health clinics primarily through telehealth services administered from VA regional hub sites to local VA clinics within the VA regional networks.^15^ While CRH was originally intended to be a site-to-site telehealth model, the majority of CRH services shifted to a site-to-home telehealth model at the onset of the pandemic.

We hypothesized that adoption of the CRH program would provide telehealth infrastructure advantages that would facilitate primary care utilization during the pandemic and also potentially reduce downstream emergency and inpatient care use as some care may be preventable through timely and improved access to primary care.^18–20^ In this study, we evaluated the impact of the pandemic on CRH’s delivery of primary care (total and by modality) and use of emergency and inpatient care within VA by comparing CRH sites and non-program sites pre- and post-pandemic.

## INTERVENTION

CRH uses a “hub-and-spoke” model to connect patients at outpatient “spoke” sites with clinical staff at “hub” sites in regional VA networks primarily via telehealth. VA’s interdisciplinary primary care teams, called Patient Aligned Care Teams (PACT), in hub sites assume responsibility for a panel of patients at spoke sites. Spoke sites with PACT team shortages and difficulties with provider recruitment may be eligible for CRH. CRH-PC sites were defined by the CRH program as sites with at least 10 CRH-PC visits per month for 2 consecutive months.

## DATA AND METHODS

### Study Cohort

To identify our study cohort, we began with all VA clinics or sites (*n* = 1110) and excluded atypical such as community living centers or sites with fewer than 450 patients over the study period, similar to prior work.^21^ The final study cohort included 1050 VA sites (CRH-PC: 254; non-CRH-PC: 796). All patients assigned to a PACT team in each site were included; patient assignments were identified from the Reengineered Patient Care Management Module in the Corporate Data Warehouse (CDW).^22^

### Outcomes

As adoption of CRH can impact both the number of care visits and patients seen at sites, we examined the quarterly number of visits and patients served per site for two types of care:

- *VA outpatient primary care*—primary care visits (total across all modalities—phone, video, and in-person and by each modality) and primary care patients served.
- *VA emergency department (ED) visits and hospitalizations*—visits to and patients seen in EDs and inpatient stays within the VA system.

We considered reporting change in visits per patient assigned to each site; however, many patients assigned to a PACT team are not expected to and do not use primary care in each quarter. We therefore reported results as the percentage change in volume of visits and patients served compared to baseline patterns of utilization, adjusting for site size. We also reported the number of visits per 1000 primary care patients served to indicate the extent of changes for patients who do utilize primary care in each quarter.

Note that if a hub provider had a telehealth visit for a patient assigned to a spoke site, the visit was attributed to the spoke site. Data on PC and ED encounters were obtained from Managerial Cost Accounting (MCA) OUT (Outpatient) National Data Extract (NDE),^23^ where we categorized primary and secondary clinic stop code pairs into categories and modalities of care. Hospitalizations were obtained from MCA TRT (Treating Specialty) NDE.^23^

### Covariates

We adjusted for differences in outcomes due to potentially differing site characteristics. We adjusted for site rurality (e.g., urban, rural, highly rural), site type (e.g., VHA Medical Center (VAMC), PC Community-Based Outpatient Clinic (PC-CBOC), multi-specialty CBOC (MS-CBOC), other), site size (number of patients with PACT team assignments by quarter), geographic region of each VA service network (East Coast, Southeast, Rocky Mountain/Gulf, Midwest, West Coast), and the quarterly average Elixhauser Comorbidity Score of patients assigned to each site. Importantly, we also included a binary indicator of whether a site ever adopted CRH-PC to adjust for any remaining unobservable differences across program and non-program sites that were time-invariant. In sensitivity analyses, we also adjusted for site-level summaries of other patient covariates such as mean age, as well as percents male, White, Black, Hispanic, and VA enrollment priorities 1 and 2.

In all models, we included the quarterly count of COVID-19 cases in each county^24^ and included quarter indicators to adjust for any shocks to health care systems or care use in each quarter, including lingering effects of the pandemic outside of the case count.

Covariate data were obtained from the CDW,^22^ VHA’s Geospatial Service Support Center,^25^ and New York Times’ COVID-19 county-year level data downloaded from GitHub.^24^

### Statistical Analyses

We first examined baseline site characteristics for CRH-PC and non-CRH-PC sites from FY2020Q1 (October-December 2019) before pandemic-related shutdown of in-person care began. We then examined unadjusted trends of CRH-PC and non-CRH-PC sites before and after the onset of COVID-19 to determine whether a difference-in-difference (DiD) framework was appropriate. Event studies improve on the traditional DiD estimator^26–28^ because they estimate differences between treatment and control group for each period prior to and after treatment (i.e., each quarter pre- and post-pandemic onset in our case).^26, 28^ This allows to visually and more transparently assess whether pre-pandemic model-adjusted differences between CRH-PC sites and non-CRH-PC sites were significant or trending upward or downward in a manner that could obscure or mask true differences in the *post*-pandemic onset period.^26, 28, 29^ An absence of pre-pandemic differences across CRH-PC and non-CRH-PC sites after covariate adjustment followed by abrupt differences post-pandemic signals attributability of findings to the pandemic.^26, 28, 29^ We also generated traditional DiD estimates to obtain the average effect of CRH-PC across all post-pandemic onset quarters (methods details in Appendix Section A).

As effects may vary across types of VA sites or site size, we conducted identical analyses, stratified by site type as site size is vastly different across site types and among these stratified analyses, further adjusted for site size. We also examined the number of visits per 1000 primary care patients served.

To strengthen attributability of findings to the CRH program, we conducted sensitivity analyses restricting the sample of CRH-PC sites to sites with pre-pandemic program implementation and to sites with program implementation during the majority of the post-pandemic onset period. We also examined reliance on CRH-PC services at CRH-PC sites (Appendix Section D).

All statistical analyses were conducted in Stata 17.0 (StataCorp, LLC).

This study followed the Strengthening the Reporting of Observational Studies in Epidemiology (STROBE) guidelines. It was funded by VA’s Office of Primary Care for quality improvement purposes and was therefore exempted from review by the Stanford institutional review board.

## RESULTS

### Unadjusted Baseline Characteristics and Trends

We found no differences in site rurality and average Elixhauser comorbidity score of patients across CRH-PC and non-CRH-PC sites, but noted some differences in site type, site size, and geographic region (Table 1). We found that VAMCs, MS-CBOCs, and larger sites were more likely to adopt CRH-PC. West Coast VA sites, followed by Rocky Mountain/Gulf region sites, were more likely to adopt CRH-PC. Similar comparisons stratified by site type are also provided in Appendix Tables 5, 6, and 7.

**Table 1:** Unadjusted Baseline Characteristics for Clinical Resource Hub Program Sites and Non-program Comparison Sites, FY2020Q1

	CRH-PC site	Non-CRH-PC site	*P*-value
*N*	254	796	
Site rurality			0.24
Urban	160 (63.0%)	478 (60.1%)	
Rural/highly rural	92 (36.2%)	299 (37.6%)	
Other/unknown	2 (0.8%)	19 (2.4%)	
Site type			< 0.001
VA Medical Center	59 (23.2%)	106 (13.3%)	
Primary Care CBOC	111 (43.7%)	405 (50.9%)	
Multi-Specialty CBOC	64 (25.2%)	138 (17.3%)	
Other/unknown	20 (7.9%)	147 (18.5%)	
Site size (number of patients with PACT team assignments in the study period), mean (SD)	9096.2 (7893.6)	5732.5 (6413.3)	< 0.001
Region			< 0.001
East Coast	46 (18.1%)	206 (25.9%)	
Southeast	30 (11.8%)	151 (19.0%)	
Rocky Mountain/Gulf	54 (21.3%)	133 (16.7%)	
Midwest	52 (20.5%)	201 (25.3%)	
West Coast	72 (28.3%)	105 (13.2%)	
Elixhauser Comorbidity Score, mean (SD)	1.2 (0.3)	1.2 (0.3)	0.96

*P*-values are derived from bivariate analyses using t-tests for continuous variables and chi-square tests for categorical variables

**Figure 1:**
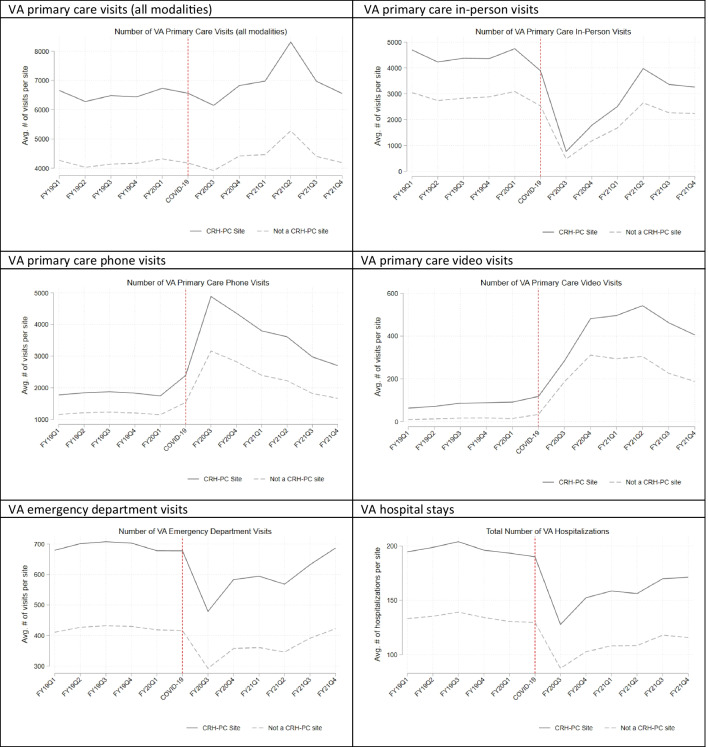
Unadjusted visit trends for VA primary care (total and by modality), emergency and inpatient care in Clinical Resource Hub program sites and non-program comparison sites FY2019–2021.

Unadjusted trends in primary care visits, ED visits, and hospitalizations show that prior to pandemic onset, CRH-PC sites and non-CRH-PC sites had roughly parallel trends, providing support for the use of DiD methods (Fig. 1). Unadjusted trends were very similar when we examined total number of patients served (Appendix Fig. 3).

### Adjusted Event Study Results

**Figure 2:**
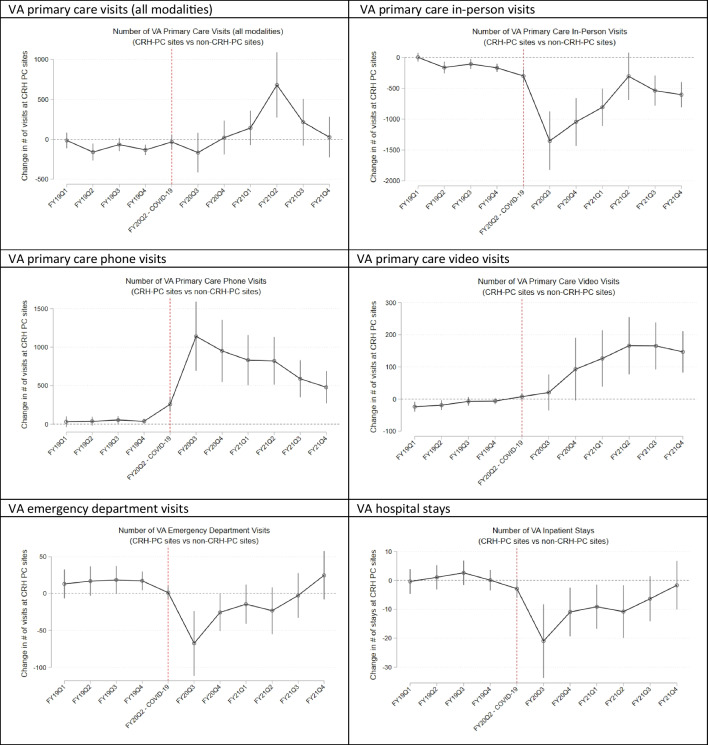
Adjusted visits for VA primary care (total and by modality), emergency and inpatient care, FY2019–FY2021, in Clinical Resource Hub program sites compared to non-program sites relative to the baseline quarter FY2020Q1—event study estimates (and 95% C.I.s).

Event study graphs show that after covariate adjustment, there were virtually no differences between CRH-PC and non-CRH-PC sites prior to the pandemic onset, whereas there were abrupt differences across CRH-PC and non-CRH-PC sites just after the onset of the pandemic (Fig. 2). This pattern of no differences pre-pandemic onset followed by abrupt differences post-onset signals attributability of post-onset changes to the pandemic onset. Event study results were very similar when we examined number of patients served (Appendix Fig. 4).

We also found similar results in sensitivity analyses restricting the sample of CRH-PC sites to sites pre-pandemic program implementation (Appendix Fig. 5) and to sites with program implementation for a majority of the post-pandemic onset period (Appendix Fig. 6). In analyses focusing on CRH-PC sites only, we demonstrated that reliance on CRH-PC increases during the post-pandemic onset period but that sites were using CRH infrastructure also for non-CRH services (Appendix Fig. 7).

### Adjusted difference-in-difference results

In traditional DiD analyses (Table 2), we found that CRH-PC sites, compared with non-CRH-PC sites, had 221 (95% CIs 11:431) additional primary care visits across all modalities per quarter, a 3.4% increase compared to prior to the pandemic. By *modality*, we found that CRH sites had 643 (95% CIs − 910: − 377) fewer in-person visits (− 14.4% compared to pre-pandemic utilization at CRH-PC sites) but had 723 (95% CIs 433:1,014) and 128 (95% CIs 56:199) more visits via phone and video telehealth per quarter (+ 39.9% and + 159.5% compared to pre-pandemic utilization) than comparison sites, respectively. We also found that CRH-PC sites had 29 (95% CIs − 57: − 1) fewer VA ED visits and 10 (95% CIs − 18: − 2) fewer VA hospital stays post-pandemic onset per quarter (− 4.2% and − 5.1% compared to pre-pandemic), respectively, compared with non-CRH-PC sites. DiD estimates were broadly similar when we examined the impact of CRH-PC on the *number of patients served* (Table 3).

**Table 2:** DiD Estimates (and 95% C.I.s) for Difference in Total Number of Visits in Clinical Resource Hub Program Sites Compared to Non-program Sites Post-pandemic

	Primary care, all modalities	In-person primary care	Telephone primary care	Video primary care	Emergency department	Inpatient stays
All sites (CRH-PC *N* = 254; non-CRH-PC *N* = 796)						
CRH × PostCOVID	220.9*	− 643.3*	723.3*	127.6*	− 29.2*	− 10.1*
	(10.9 430.9)	(− 909.9 − 376.7)	(432.5 1014.1)	(55.9 199.2)	(− 57.2 − 1.1)	(− 18.1 − 2.1)
% Change	3.4%	− 14.4%	39.9%	159.5%	− 4.2%	− 5.1%
VAMCs (CRH-PC *N* = 59; non-CRH-PC *N* = 106)						
CRH × PostCOVID	− 149.7	− 260.6	56.4	94.7	9.5	12.9
	(− 964.8 665.3)	(− 1128.3 607.0)	(− 932.0 1044.8)	(− 142.0 331.4)	(− 102.9 121.9)	(− 16.6 42.3)
% Change	− 1.2%	− 3.0%	1.6%	119.9%	0.5%	2.4%
PC-CBOCs (CRH-PC *N* = 111; non-CRH-PC *N* = 405)						
CRH × PostCOVID	125.9	− 240.1*	300.2*	54.6	− 1.3	− 1.3
	(− 32.1 283.8)	(− 402.9 − 77.2)	(107.0 493.4)	(− 13.3 122.5)	(− 15.9 13.2)	(− 4.8 2.2)
% Change	3.7%	− 10.4%	31.6%	70.9%	− 0.6%	− 2.1%
MS-CBOCs (CRH-PC *N* = 64; non-CRH-PC *N* = 138)						
CRH × PostCOVID-	521.4*	− 478.0	915.3*	65.3	− 10.8	− 5.1
	(112.9 929.8)	(− 968.1 12.2)	(283.3 1547.4)	(− 77.9 208.5)	(− 47.0 25.4)	(− 16.2 5.9)
% Change	7.1%	− 9.4%	46.4%	76.7%	− 2.2%	− 3.1%

*Results that were statistically significant, with *P*-value < 0.05. Standard errors were adjusted for clustering by site

**Table 3:** DiD Estimates (and 95% C.I.s) for Patients with Each Type of Care in Clinical Resource Hub Program Sites Compared to Non-program Sites Post-pandemic

	Primary Care, all modalities	In-person Primary Care	Telephone Primary Care	Video Primary Care	Emergency Department	Inpatient Stays
All sites (CRH-PC *N* = 254; non-CRH-PC *N* = 796)						
CRH × PostCOVID	19	− 457*	423*	115*	− 23*	− 8*
	(− 45 83)	(− 636 − 278)	(262 583)	(52 177)	(− 41 − 4)	(− 14 − 3)
% Change	0.5%	− 14.3%	37.4%	166.7%	− 4.6%	− 5.7%
VAMCs (CRH-PC *N* = 59; non-CRH-PC *N* = 106)						
CRH × PostCOVID	4	− 290	165	89	1	5
	(− 238 246)	(− 835 256)	(− 354 684)	(− 114 292)	(− 74 75)	(− 14 23)
% Change	0.1%	− 4.7%	7.5%	125.4%	0.1%	1.3%
PC CBOCs (CRH-PC *N* = 111; non-CRH-PC *N* = 405)						
CRH × PostCOVID	18	− 162*	177*	50	− 2	− 1
	(− 24 60)	(− 277 − 48)	(74 280)	(− 10 111)	(− 12 8)	(− 4 1)
% Change	0.9%	− 9.7%	29.6%	75.8%	− 1.2%	− 2.2%
MS-CBOCs (CRH-PC *N* = 64; non-CRH-PC *N* = 138)						
CRH × PostCOVID	99	− 340	481*	59	− 8	− 4
	(− 1 200)	(− 682 2)	(130 832)	(− 69 187)	(− 33 17)	(− 12 3)
% Change	2.4%	− 9.2%	38.7%	81.9%	− 2.2%	− 3.4%

*Results that were statistically significant, with *P*-value < 0.05. Standard errors were adjusted for clustering by site

Examining the number of visits per 1000 primary care patients served (Table 4), we did not find significant differences in in-person visits or video visits per patient served, but found that CRH-PC sites had 48 (95% CIs 10: 85) additional phone primary care visits per 1000 primary care patients served, compared to comparison sites.

**Table 4:** DiD Estimates (and 95% C.I.s) for Differences in Number of Visits per 1000 Primary Care Patients Served in Clinical Resource Hub Program Sites Compared to Non-program Sites Post-pandemic

	Primary care, all modalities	In-person primary care	Telephone primary care	Video primary care	Emergency department	Inpatient stays
CRH × PostCOVID	6.8	− 32.3	47.9*	− 10.2	− 2.2	1.0
	(− 29.2 42.7)	(− 65.2 0.5)	(10.4 85.4)	(− 26.3 5.9)	(− 8.4 4.0)	(− 3.5 5.5)
% Change	0.4%	− 2.7%	9.8%	− 20.3%	− 1.6%	2.3%

*Results that were statistically significant, with *P*-value < 0.05. Standard errors were adjusted for clustering by site

In analyses stratified by site type (Table 2), we found that PC-CBOCs with CRH-PC, compared to PC-CBOCs without CRH-PC, had 240 fewer in-person visits (− 10.4% compared to pre-pandemic) but 300 additional phone visits (+ 31.6%). MS-CBOCs with CRH-PC, compared to MS-CBOCs without CRH-PC, had additional 521 primary care visits across all modalities (+ 7.1%) and 915 more phone visits for primary care (+ 46.4%). Examining the number of patients, we found that PC-CBOCs and MS-CBOCs with CRH served more patients through telephone visits than comparison PC-CBOCs and MS-CBOCs (+ 29.6% and + 38.7%, respectively, compared to pre-pandemic) (Appendix, Table C1).

## DISCUSSION

This study is the first to compare use of primary care in sites with and without CRH-PC before and after the COVID-19 pandemic. We found that VA’s CRH-PC program was associated with a modest 3.4% increase in primary care visit volume across all modalities (in-person, telephone, video, and other) during the pandemic, driven by increases in telephone and video visits, which offset declines in in-person visits that occurred immediately after the onset of the pandemic. We also found that CRH-PC was associated with modest decreases in VA ED visits and in VA inpatient stay volumes (− 4.6% and − 5.7%, respectively) relative to non-CRH-PC sites. Findings were similar when we examined the volume of patients served in primary care.

We stratified all our analyses by site type as medical center-based clinics are typically much larger than community-based outpatient clinics. In these analyses, we found that the pattern of volume declines in in-person visits and offsetting increases in telehealth for primary care was more prominent at VA’s PC-CBOCs and MS-CBOCs. This is consistent with prior studies on CRH (and its pilot versions^16, 17^) which have shown that over 53% of CRH care was delivered at MS-CBOCs with CRH’s overall intention and focus being to serve such under-resourced CBOCs, many of which are in geographic areas where substantial numbers of Veterans reside but these areas face healthcare professional shortages.^15^

When we examined the extent of visits among patients who utilized primary care in each quarter, we found that CRH-PC sites, compared to non-program sites, had 48 additional telephone visits per 1000 primary care patients served (+ 9.8%).

This study contributes to several strands of literature. Firstly, it builds upon recent work evaluating VA’s CRH, providing critical quantitative evidence to complement qualitative work suggesting that VA’s CRH facilitated health system resiliency.^11^ Our comparison of CRH-PC vs. non-CRH-PC sites expands on prior analyses focusing on CRH-only visits^30^ to strengthen the link between VA’s CRH program and facilitation of primary care visits during the COVID-19 pandemic.

Next, we contribute to the literature on improving access to primary care via telehealth. Similar to prior studies examining primary care visits across all modalities which typically find either no impact or modest impact on overall primary care use,^5, 16, 31^ we found VA’s CRH-PC to be associated with a modest 3.4% increase in primary care visits across all modalities, a magnitude closely aligned with a recent large-scale study from Israel showing pandemic-driven access to telehealth increased primary care visits by 3.5%.^31^

Finally, we found that CRH-PC adoption was associated with very modest decreases in the volume of VA ED visits and hospital stays during the pandemic. However, we did not have data on care provided in the community for this study despite the large growth in community care since the MISSION Act. It is possible that patients in CRH sites sought more care at EDs and hospitals outside the VA health care system during the pandemic. Therefore, the impact of CRH adoption on total acute and inpatient utilization is still unclear. Additional studies examining CRH’s impact on non-VA care are needed.

### Limitations

There are some limitations of this study. As our study focused on the impact of the CRH-PC program during the COVID-19 pandemic, we did not examine utilization pre-post CRH-PC adoption at sites as we did not have site-specific start dates for CRH. As such, it is possible that some CRH sites continued to adopt CRH-PC post-pandemic onset. Analyses focusing on the association with the specific timing of uptake or penetration of CRH at VA sites would be helpful for further isolating the impact of CRH. Nonetheless, in sensitivity analyses restricting the sample of CRH-PC sites to sites that adopted CRH-PC pre-pandemic (Fig. 5 in the Appendix) or sites that had CRH-PC during the majority of the post-pandemic period (Fig. 6 in the Appendix), we observed very similar results.

Next, an unavoidable methodological limitation in evaluating a population-level intervention is there may have been unobservable factors that influenced outcomes and the adoption of CRH-PC. As such, we leveraged a DiD design which allows for *level* differences in baseline characteristics across CRH-PC and comparison sites. DiD allows for unobservable reasons for program adoption as long as CRH-PC and comparison sites exhibit parallel trends in outcomes. We first provided evidence that unadjusted trends exhibit parallel outcome trends for CRH-PC and non-CRH-PC sites (Fig. 1), and then in adjusted event study graphs (Fig. 2), we demonstrated that pre-pandemic differences observed between CRH-PC and non-CRH-PC sites were eliminated after covariate adjustment. The stark contrast in differences across CRH-PC and non-CRH-PC sites immediately post-pandemic then strengthened attributability of post-pandemic differences to CRH-PC. Nonetheless, our methods are not able to distinguish between differences in CRH-PC and non-CRH-PC sites unrelated to the CRH program which may have occurred simultaneously with pandemic onset. For example, if CRH-PC sites improved their infrastructure or management in response to the pandemic for reasons unrelated to the CRH program, our analysis cannot disentangle these associations from the associations with the CRH program. Additional studies of CRH will be important to validate our findings.

We also did not study health care utilization that may have occurred outside VA so utilization by patients in CRH and non-CRH sites may have been under-measured. Additional studies are needed to examine the impact of CRH on total outpatient, acute, and inpatient care for each patient. Furthermore, as CRH also provided mental health care and specialty care, our analyses focusing on primary care remain limited in scope. Future work should also examine CRH’s impact on these other types of care. Finally, our results may not readily generalize to all types of telehealth programs or to non-VA settings.

## CONCLUSION

VA’s pre-pandemic rollout of the Clinical Resource Hub telehealth program for primary care intended to improve health care access at under-resourced clinics facilitated primary care during the pandemic, a period fraught with care disruptions and limited in-person health care delivery. Telehealth may be an important strategy to maintain access to care and offer health system resilience during times of crises.

### Supplementary Information

Below is the link to the electronic supplementary material.

Supplementary file1 (DOCX 1146 KB)

## Data Availability

Data cannot be shared publicly because of VA policies regarding data privacy and security. Data contain potentially identifying and sensitive patient information. All relevant de-identified data are included in the manuscript. For investigators with appropriate authorizations within the Department of Veterans Affairs, contact HERC@va.gov or VINCI@va.gov for more information about accessing data.

## Declarations

### Disclaimer

The funders were not involved in the design and conduct of the study, interpretation of the data, preparation, and review of this manuscript, nor had a role in the decision to submit the manuscript for publication. We are also grateful for our discussions with Adam Chow, BA, Chelle L. Wheat, PhD, and Steve Fihn, MD, MPH about this manuscript.
