# Supplementary material for: The Clinical Resource Hub Telehealth Program and Use of Primary Care, Emergency, and Inpatient Care During the COVID-19 Pandemic
Source: J Gen Intern Med. 2024 Jan 22;39(Suppl 1):118–26. doi: 10.1007/s11606-023-08476-x (PMC10937884; doi:10.1007/s11606-023-08476-x)
Supplement: Supplementary file 1 — Supplementary file1 (DOCX 1146 KB) [file 11606_2023_8476_MOESM1_ESM.docx]

# Appendix - The Clinical Resource Hub telehealth program and use of primary care, emergency, and inpatient care during the COVID-19 Pandemic

Contents

[A. Statistical Methods Details 1](#_Toc142054940)

[B. Supplementary Results – Unadjusted baseline characteristics for Clinical Resource Hub program sites and non-program comparison sites, stratified by facility type 2](#_Toc142054941)

[C. Supplementary Results – Total Number of Patients Using Each Type of Care in a Site 3](#_Toc142054942)

[D. Sensitivity Analyses – Total Number of Visits for Each Type of Care, restricting the sample of sites in each analysis 6](#_Toc142054943)

## Statistical Methods Details

We first examined baseline site characteristics for CRH-PC and non-CRH-PC sites from FY2020Q1 (October – December 2019) before pandemic-related shutdown of in-person care began. We then examined unadjusted trends of CRH-PC and non-CRH-PC sites before and after the onset of COVID-19 to assess parallel trends in outcomes prior to the pandemic to determine whether a difference-in-difference (DiD) framework was appropriate, as DiD designs assume that treatment (CRH-PC sites) and control (non-CRH-PC sites) groups would have exhibited similar or parallel trends in the absence of treatment (CRH-PC). Recent developments show that event studies improve on the usual DiD estimator because they provide DiD estimates for each period (each quarter in this study) prior to and after treatment (i.e. pre- and post-pandemic onset in this study). This allows for visually and more transparently assessing whether pre-pandemic model-adjusted differences between CRH-PC sites and non-CRH-PC sites were significant or trending upward or downward in a manner that could obscure or mask true differences in the post-pandemic period. These adjusted pre-pandemic trends provide important context for interpreting post-pandemic onset differences. An absence of differences across CRH-PC and non-CRH-PC sites prior to the pandemic after covariate adjustment followed by abrupt differences post-pandemic signals that post-pandemic differences can be attributed to the pandemic onset. Finally, we estimated differences in outcomes using traditional DiD analyses to provide an estimate of the average effect of CRH-PC across all post-pandemic onset quarters. We used linear regression models with robust standard errors clustered by spoke site for the event study and the traditional difference-in-difference). As effects may differ across the different types of VA sites or based on site size, we conducted identical analyses, stratified by clinic or site type and also examined the number of visits per 1,000 primary care patients served in a site.

The event study specification was as follows:

$$Y_{i,t}= \beta_{0} + \beta_{1}{CRHPC}_{i}+ \beta_{2}{QuarterRelativeToCOVID19onset}_{i,t}+ \beta_{3-13}CRHPC*{QuarterRelativeToCOVID19onset}_{i,t}+\beta_{14}{SiteCharacteristics}_{i,t}+ \varepsilon$$

where $Y_{i,t}$ is the outcome or dependent variable of interest, *i* denotes a VA spoke site, and *t* denotes fiscal year quarters. $CRHPC$ is a binary indicator for whether a spoke site adopted CRH-PC and captures the fixed or time-invariant difference between CRH-PC sites and non-CRH-PC sites. As pandemic-related shutdown of in-person care began in March 2020 or FY20Q2, we considered FY20Q1 as the baseline quarter. $\beta_{2}$, the coefficient for the variable *QuarterRelativeToCOVID19onset,* captures the quarterly variation in outcomes, relative to the baseline quarter. The primary variable of interest was the interaction term, *CRHPC* QuarterRelativeToCOVID19onset* and $\beta_{3-13}$were the coefficients of interest, which reflect the difference in outcomes at CRH-PC vs. at non-CRH-PC sites each quarter pre- and post-pandemic onset, compared to outcomes at these sites in the baseline quarter of FY20Q1. These coefficients were plotted on the event study graphs to illustrate the effects of the CRH-PC program in the pre- and post-pandemic periods. $\beta_{14}$ captures the outcome associations with site-level characteristics we adjusted for such as site type, site rurality, geographic region, and the number of COVID-19 cases in each county per quarter where a site was located. $\varepsilon$ denotes the error term.

We then conducted a traditional DID estimation using the following specification:

$$Y_{i,t}= \beta_{0}+\beta_{1}{CRHPC}_{i}+ \beta_{2}{PostCOVID19onset}_{t}+{\beta_{3}CRH*{PostCOVID19onset}_{i,t}+ \beta}_{4}{SiteCharacteristics}_{i,t}+ \varepsilon$$

where *PostFY20Q1* is a binary indicator for whether an observation is from the post-pandemic onset period or from the pre-pandemic period. *PostCOVID19onset* takes on a value of 1 after FY20Q2 and 0 otherwise. *CRHPC* PostCOVID19onset* is the primary variable of interest. $\beta_{3}$, which is the coefficient for the interaction term, *CRHPC*PostCOVID19onset*, represents the average effect of the CRH-PC program across all the post-pandemic onset quarters in the study.

The key difference between the event study and the traditional DID specification is that the DID estimation aggregates or averages the differences between CRH-PC sites and non-CRH-PC sites in all post-pandemic quarters and compares it to the average pre-pandemic difference in outcomes, whereas the event study provides estimated differences across CRH-PC and non-CRH-PC sites *in each quarter*, compared to the difference between these sites at baseline, in FY20Q1.

## Supplementary Results – Unadjusted baseline characteristics for Clinical Resource Hub program sites and non-program comparison sites, stratified by facility type

Table B1. Unadjusted Baseline Characteristics in FY20Q1 for Clinical Resource Hub Program VA Medical Centers (VAMCs) and non-program VAMCs.

| **Site Characteristics** | **CRH-PC VAMC** | **Non-CRH-PC VAMC** | **P-value** |
| --- | --- | --- | --- |
| N | 59 | 106 |  |
| Site rurality |  |  | 0.47 |
| Urban | 52 (88.1%) | 89 (84.0%) |  |
| Rural/highly rural | 7 (11.9%) | 17 (16.0%) |  |
| Site size (Number of patients assigned to a primary care PACT team), mean (SD) | 18048.0 (8870.1) | 15285.4 (10072.1) | 0.080 |
| Region |  |  | 0.028 |
| East Coast | 12 (20.3%) | 36 (34.0%) |  |
| Southeast | 7 (11.9%) | 19 (17.9%) |  |
| Rocky Mountain-Gulf | 9 (15.3%) | 16 (15.1%) |  |
| Midwest | 14 (23.7%) | 24 (22.6%) |  |
| West Coast | 17 (28.8%) | 11 (10.4%) |  |
| Elixhauser comorbidity score, mean (SD) | 1.4 (0.3) | 1.6 (0.3) | <0.001 |

*P-values are derived from bivariate analyses using t-tests for continuous variables and chi-square tests for categorical variables.*

Table B2. Unadjusted Baseline Characteristics in FY20Q1 for Clinical Resource Hub Program Primary Care Community Based Outpatient Clinics (PC-CBOCs) and non-program PC-CBOCs.

| **Site Characteristics** | **CRH PC-CBOCs** | **Non-CRH PC-CBOCs** | **P-value** |
| --- | --- | --- | --- |
| N | 111 | 405 |  |
| Site rurality |  |  | 0.24 |
| Urban | 52 (46.8%) | 226 (55.8%) |  |
| Rural/highly rural | 57 (51.4%) | 172 (42.5%) |  |
| Other/unknown | 2 (1.8%) | 7 (1.7%) |  |
| Site size, mean (SD) | 5178.0 (3640.9) | 3748.3 (2713.0) | <0.001 |
| Region |  |  | <0.001 |
| East Coast | 24 (21.6%) | 103 (25.4%) |  |
| Southeast | 11 (9.9%) | 90 (22.2%) |  |
| Rocky Mountain-Gulf | 25 (22.5%) | 53 (13.1%) |  |
| Midwest | 22 (19.8%) | 109 (26.9%) |  |
| West Coast | 29 (26.1%) | 50 (12.3%) |  |
| Elixhauser score, mean (SD) | 1.2 (0.3) | 1.2 (0.3) | 0.24 |

*P-values are derived from bivariate analyses using t-tests for continuous variables and chi-square tests for categorical variables.*

Table B3. Unadjusted Baseline Characteristics in FY20Q1 for Clinical Resource Hub Program Multi-Specialty Community Based Outpatient Clinics (MS-CBOCs) and non-program MS-CBOCs.

| **Site Characteristics** | **CRH-PC MS-CBOCs** | **Non-CRH-PC MS-CBOCs** | **P-value** |
| --- | --- | --- | --- |
| N | 64 | 138 |  |
| Site rurality |  |  | 0.44 |
| Urban | 50 (78.1%) | 113 (81.9%) |  |
| Rural/highly rural | 14 (21.9%) | 23 (16.7%) |  |
| Other/unknown | 0 (0.0%) | 2 (1.4%) |  |
| Site size, mean (SD) | 11153.9 (7959.6) | 8363.9 (5919.0) | 0.006 |
| Region |  |  | 0.36 |
| East Coast | 9 (14.1%) | 29 (21.0%) |  |
| Southeast | 10 (15.6%) | 23 (16.7%) |  |
| Rocky Mountain-Gulf | 15 (23.4%) | 25 (18.1%) |  |
| Midwest | 14 (21.9%) | 39 (28.3%) |  |
| West Coast | 16 (25.0%) | 22 (15.9%) |  |
| Elixhauser score, mean (SD) | 1.2 (0.3) | 1.2 (0.2) | 0.19 |

*P-values are derived from bivariate analyses using t-tests for continuous variables and chi-square tests for categorical variables.*

## Supplementary Results – Total Number of Patients Using Each Type of Care in a Site

Figure C1: Trends (unadjusted) in the number of patients with primary care (total and by modality) visits, emergency visits, and inpatient stays

| VA primary care patients (all modalities) | VA primary care in-person patients |
| --- | --- |
| 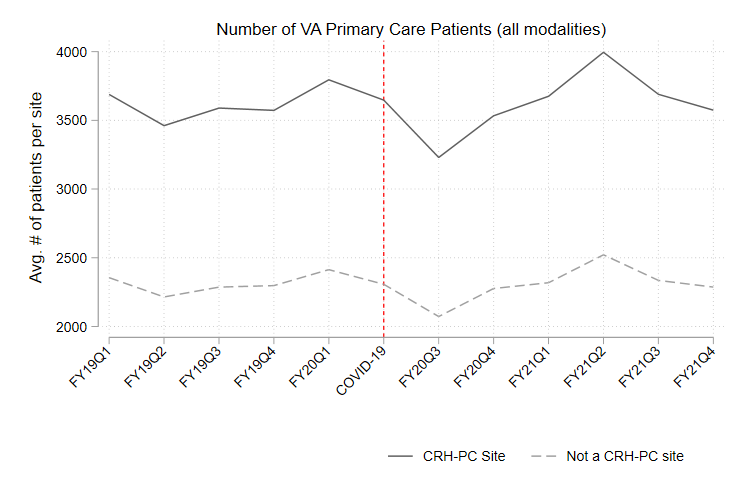 | 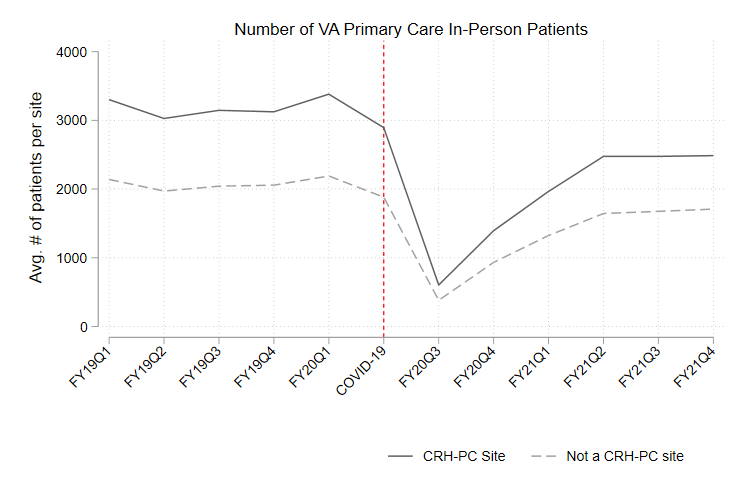 |
| VA primary care phone patients | VA primary care video patients |
| 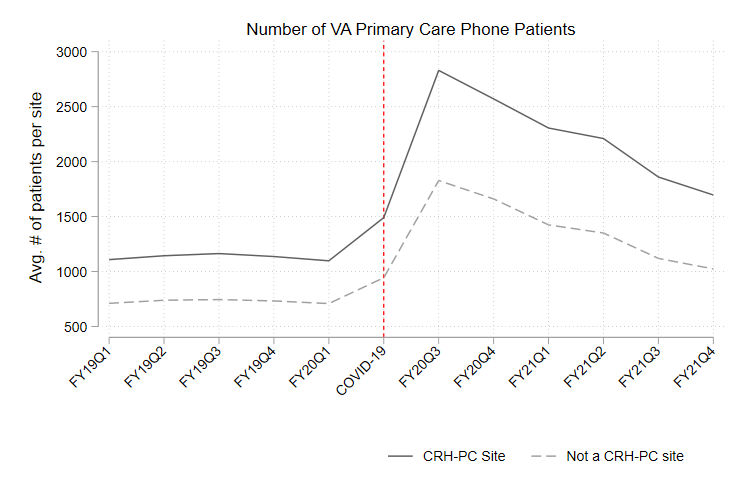 | 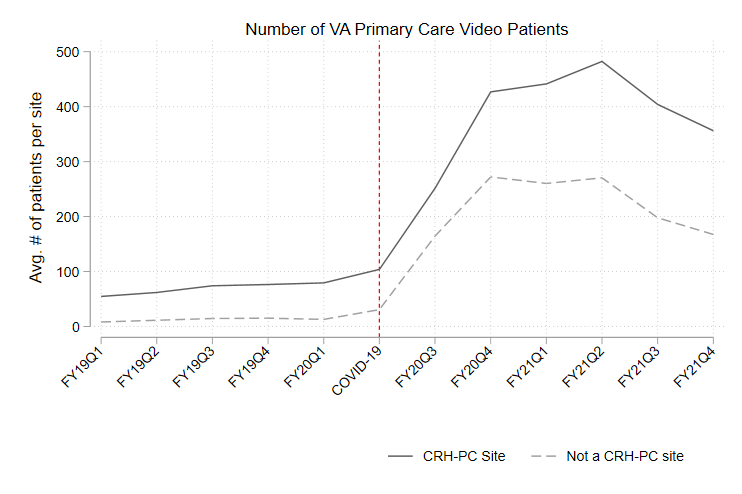 |
| VA emergency department patients | VA patients hospitalized |
| 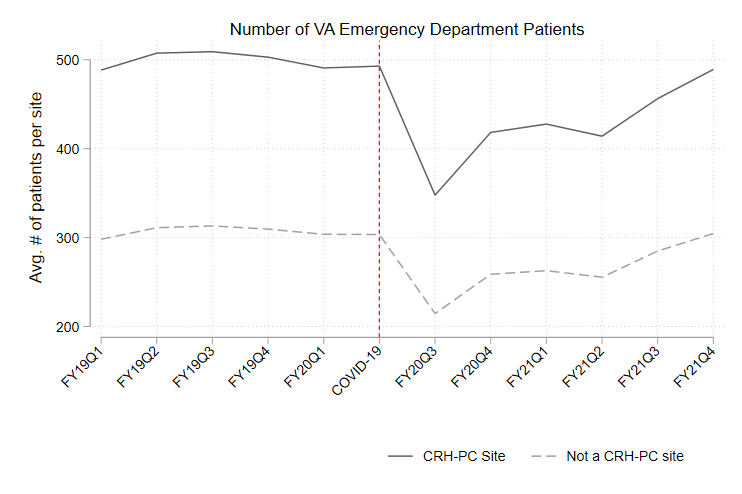 | 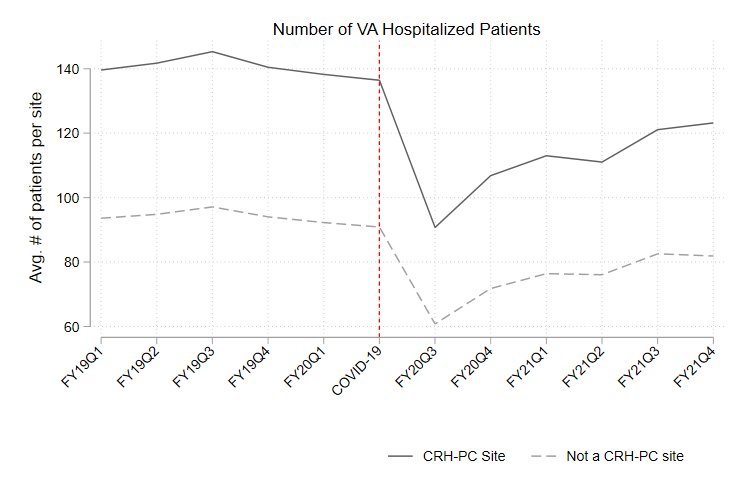 |

Figure C2: Number of patients with primary care visits, ED visits and inpatient stays in Clinical Resource Hub program sites compared to non-program sites, FY2019-FY2021, relative to the baseline quarter FY2020Q1 – adjusted event study estimates (and 95% C.I.s)

| VA primary care patients (all modalities) | | | VA primary care in-person patients | | | | |  |
| --- | --- | --- | --- | --- | --- | --- | --- | --- |
| 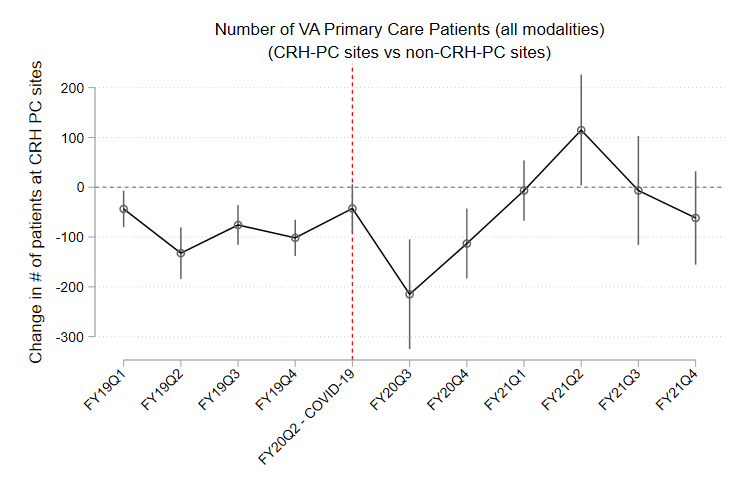 | | | 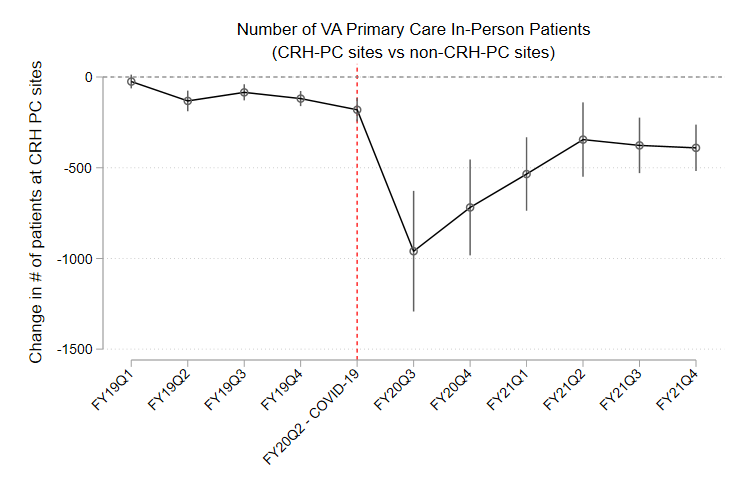 | | | | |  |
| VA primary care phone patients | | | VA primary care video patients | | | | |  |
| 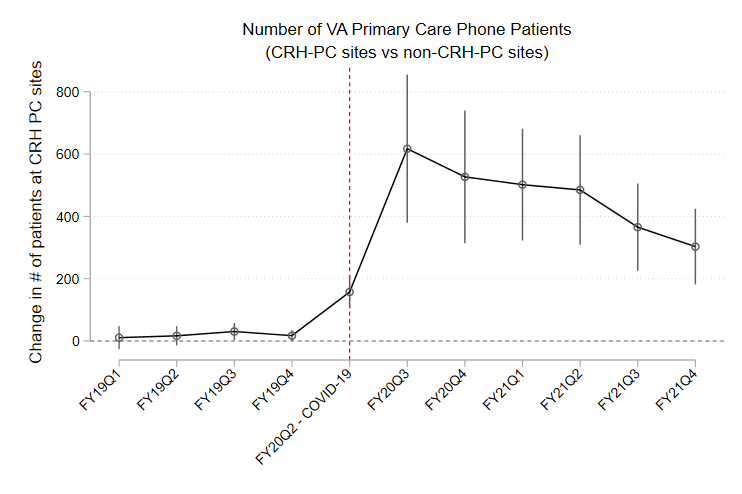 | | | 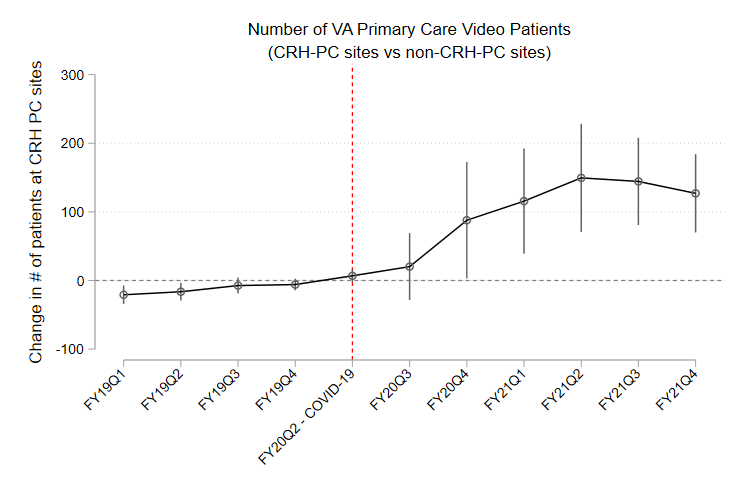 | | | | |  |
| VA emergency department patients | | | VA patients hospitalized | | | | |  |
| 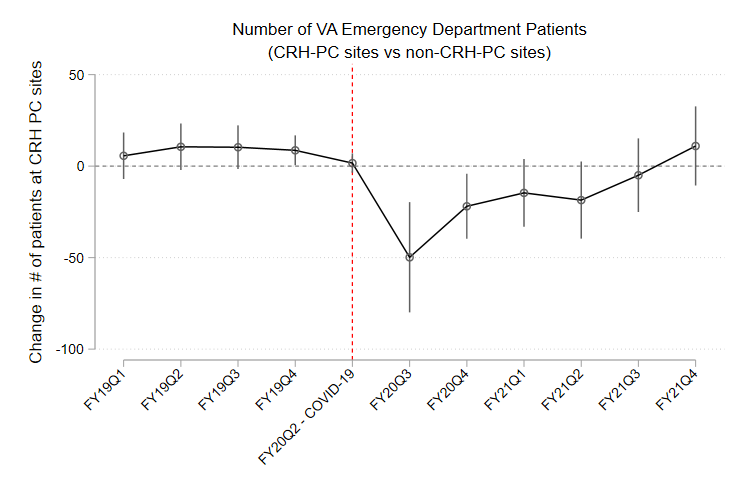 | | | 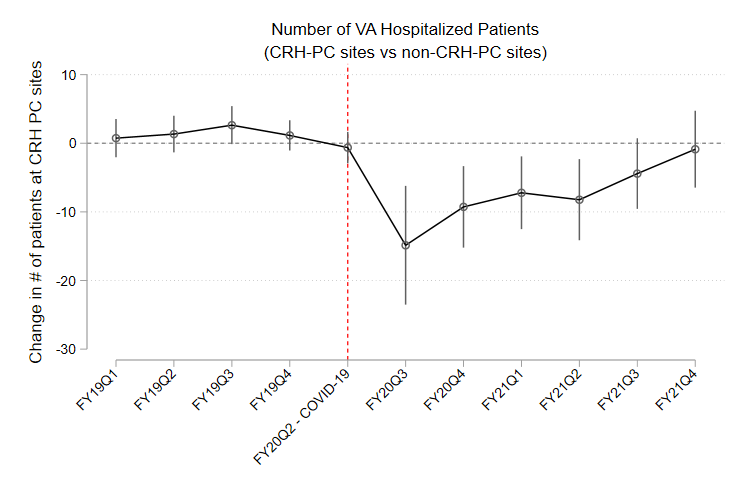 | | | | |  |
| Table C1. DID estimates (and 95% C.I.s) for difference in total number of patients served in Clinical Resource Hub program sites compared to non-program sites post-pandemic, stratified by site type | | | | | | | | |
|  | Primary Care, all modalities | In-person Primary Care | | Telephone Primary Care | Video Primary Care | Emergency Department | Inpatient Stays | |
| **VAMCs (CRH-PC N=59, Non-CRH-PC N=106)** | | | | | | | | |
| CRH*PostCOVID | 4 | -290 | | 165 | 89 | 1 | 5 | |
|  | (-238 246) | (-835 256) | | (-354 684) | (-114 292) | (-74 75) | (-14 23) | |
| % Change | 0.1% | -4.7% | | 7.5% | 125.4% | 0.1% | 1.3% | |
| **PC-CBOCs (CRH-PC N=111 , Non-CRH-PC=405)** | | | | | | | | |
| CRH*PostCOVID | 18 | -162* | | 177* | 50 | -2 | -1 | |
|  | (-24 60) | (-277 -48) | | (74 280) | (-10 111) | (-12 8) | (-4 1) | |
| % Change | 0.9% | -9.7% | | 29.6% | 75.8% | -1.2% | -2.2% | |
| **MS-CBOCs (CRH-PC N=64, Non-CRH-PC N=138)** | | | | | | | | |
| CRH*PostCOVID | 99 | -340 | | 481* | 59 | -8 | -4 | |
|  | (-1 200) | (-682 2) | | (130 832) | (-69 187) | (-33 17) | (-12 3) | |
| % Change | 2.4% | -9.2% | | 38.7% | 81.9% | -2.2% | -3.4% | |

** Asterisks indicate results that were statistically significant, with p-value <0.05*

## Sensitivity Analyses – Total Number of Visits for Each Type of Care, restricting the sample of sites in each analysis

To strengthen attributability of the primary findings to the CRH program and to offer additional insights into reliance on the CRH program, we conducted the following three sensitivity analyses:

1. We restricted CRH-PC CRH PC sites to those with program implementation before onset of COVID-19

Figure D1: CRH PC sites with pre-pandemic program implementation vs non-CRH PC sites - event study estimates (and 95% C.I.s)

| VA primary care patients (all modalities) | VA primary care in-person patients |
| --- | --- |
| 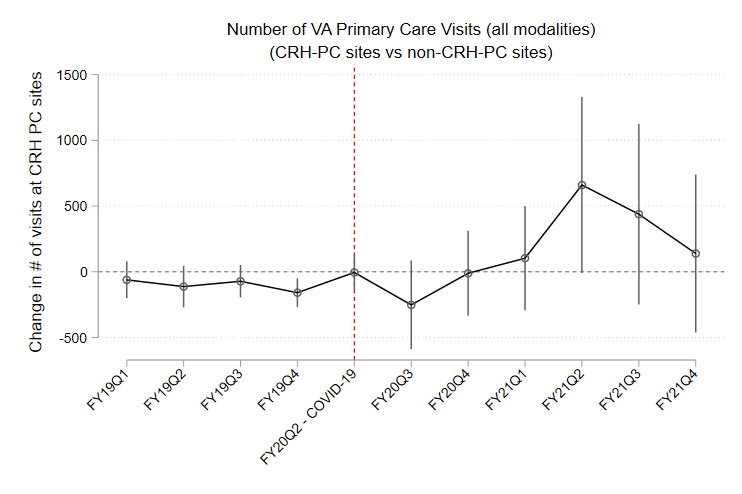 | 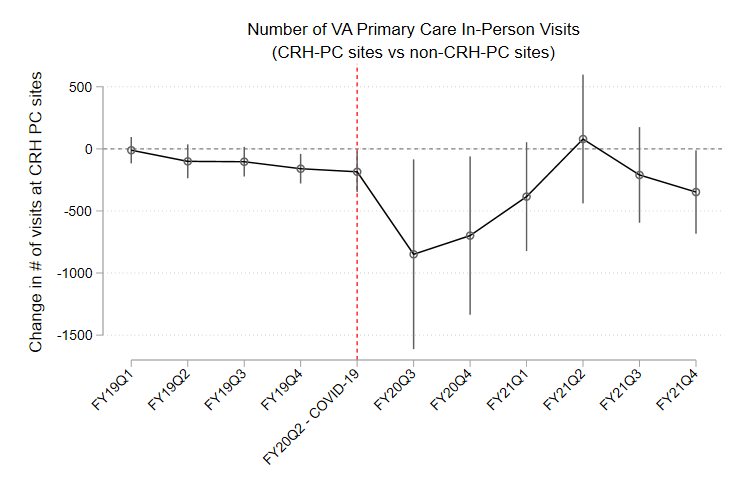 |
| VA primary care phone patients | VA primary care video patients |
| 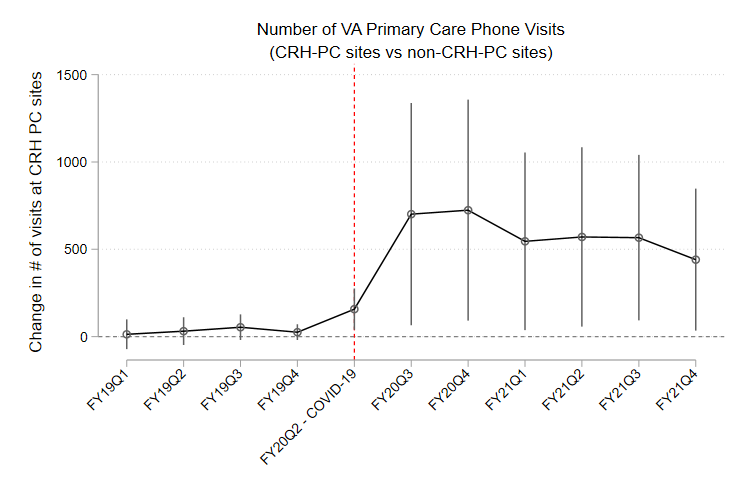 | 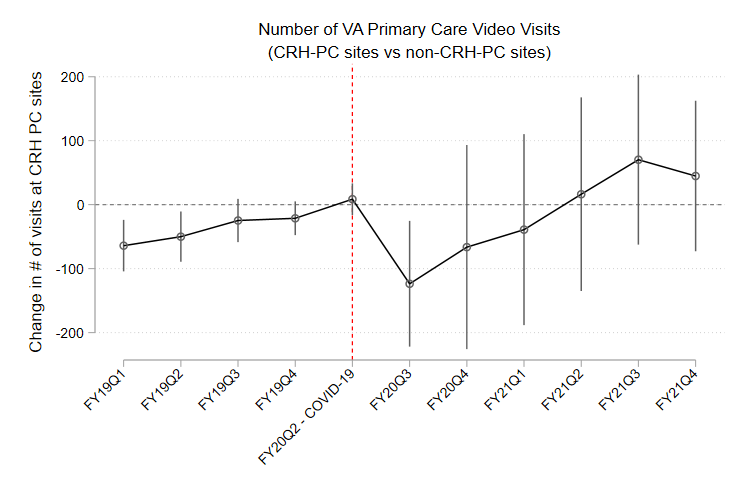 |
| VA emergency department patients | VA patients hospitalized |
| 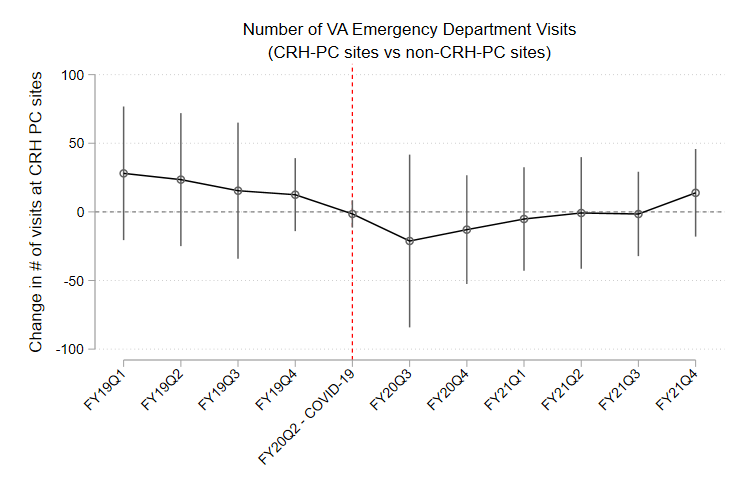 | 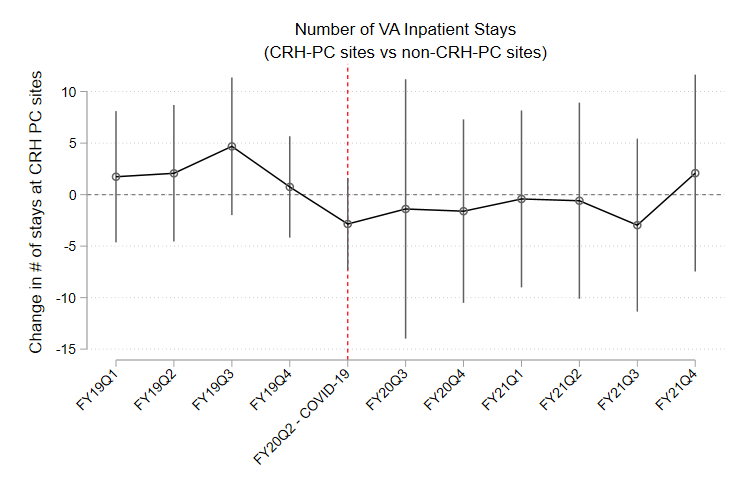 |

CRH PC sites, N = 82; Non-CRH PC sites, N = 796

1. We restricted the sample of CRH-PC sites to those implemented before 12/31/2020 to allow CRH PC implementation of 4 or more quarters during the post-pandemic period

Figure D2: CRH PC sites with 4 or more quarters of program implementation during post-pandemic period vs non-CRH PC sites– event study estimates (and 95% C.I.s)

| VA primary care visits (all modalities) | VA primary care in-person visits |
| --- | --- |
| 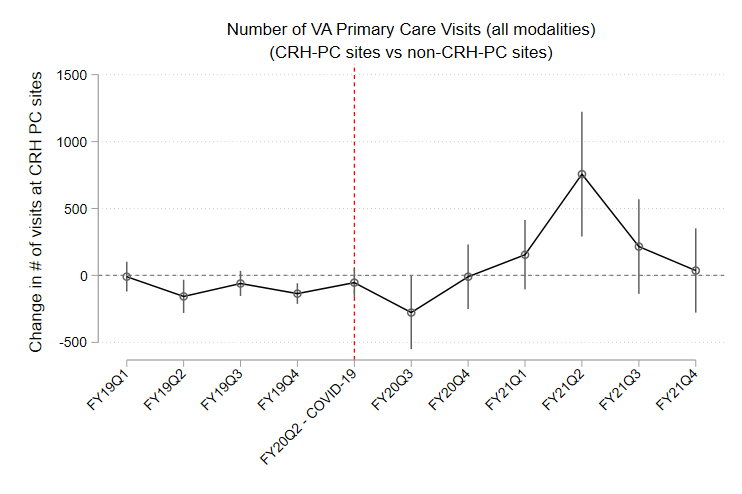 | 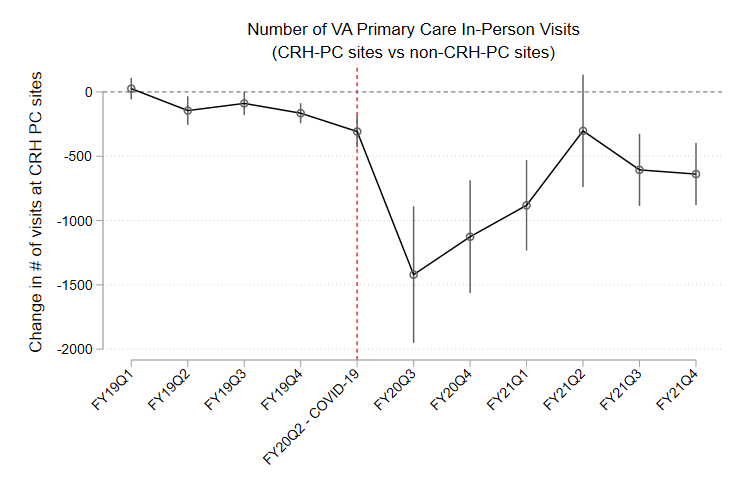 |
| VA primary care phone visits | VA primary care video visits |
| 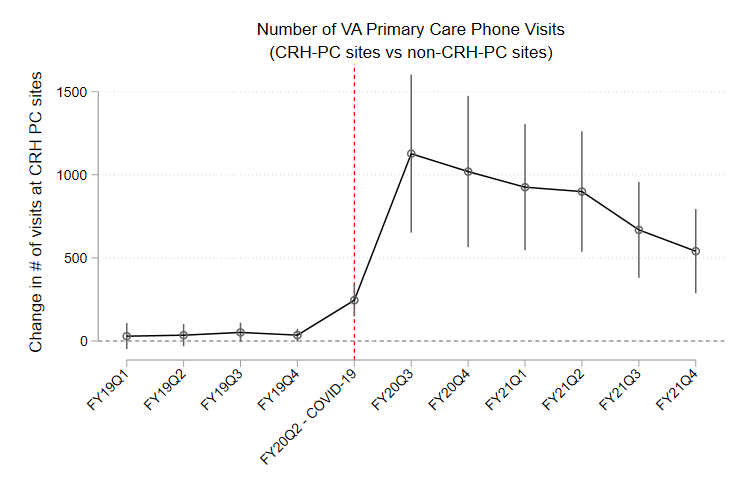 | 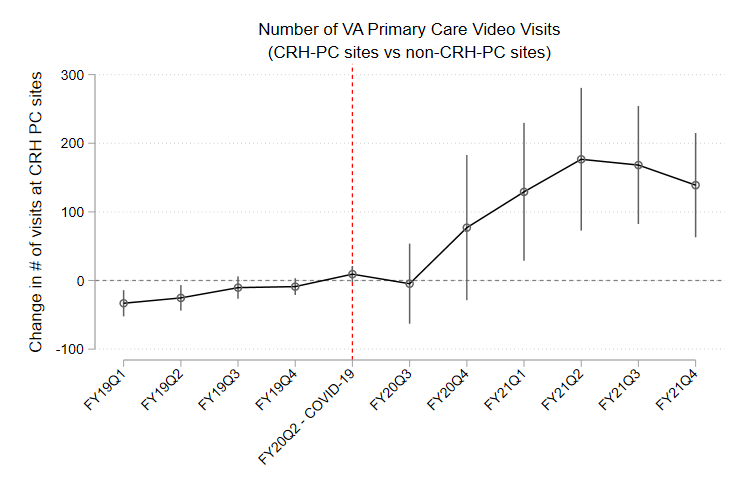 |
| VA emergency department visits | VA hospital stays |
| 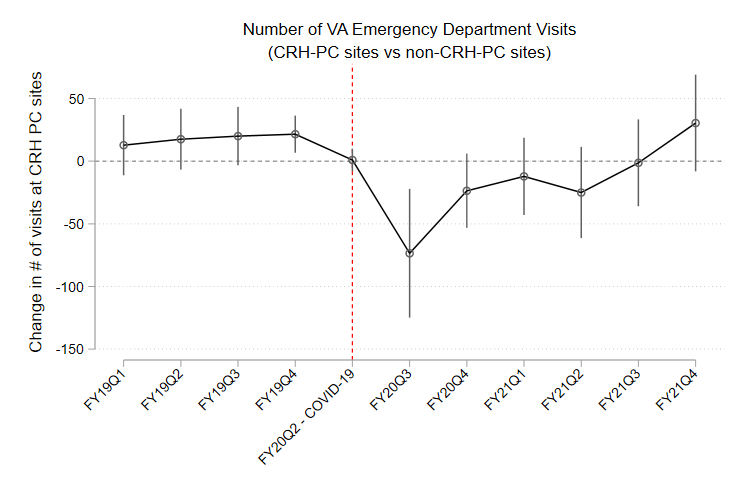 | 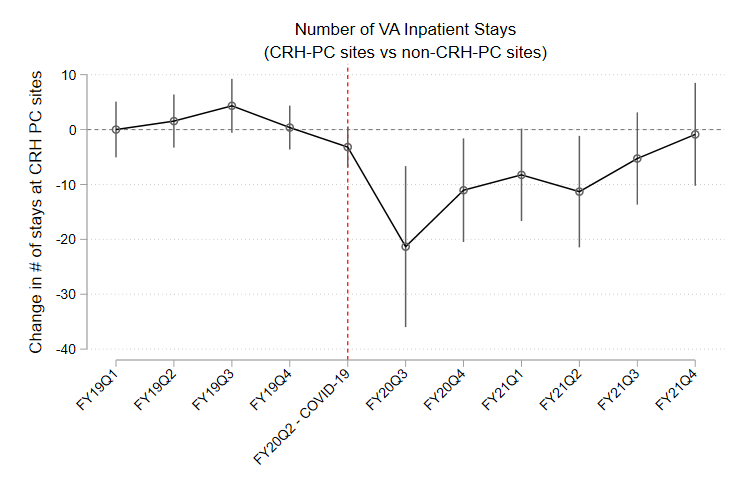 |

CRH PC sites, N = 195; Non-CRH PC sites, N = 796

1. We analyzed changes in reliance on the CRH-PC program for CRH-PC sites by examining the percentage of primary care visits (total and by modality) which occurred through the CRH program.

Figure D3 below shows that compared to FY2020 quarter 1, there was increased reliance on the CRH-PC program during the pandemic.

For primary care video visits, although we see a drop in the percentage of video visits through CRH-PC post-pandemic compared to the baseline quarter, this drop is due to the substantial increase in video visits overall (through non-CRH services) such that CRH-PC video visits were now a smaller percentage of all video primary care visits. Figure D3 shows that over time though, percentage of video visits through CRH-PC increase post-pandemic as well. Nonetheless, as each of the sub-figures in Figure D3 shows that the magnitude of reliance on CRH-PC services was less than 100%, this suggests that CRH-PC sites were likely using the telehealth infrastructure developed for CRH-PC services for non-CRH-PC services during the pandemic such that there was an overall increase in video and telephone visits due to adoption of the CRH-PC program, as shown in the manuscript.

Figure D3: CRH-PC sites only - adjusted visits relative to baseline quarter FY2020Q1– event study estimates (and 95% C.I.s)

| VA primary care visits (all modalities) | VA primary care in-person visits |
| --- | --- |
| 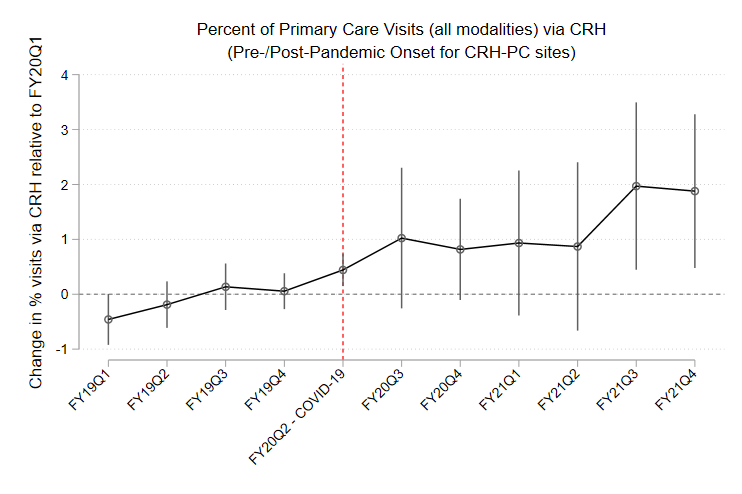 | 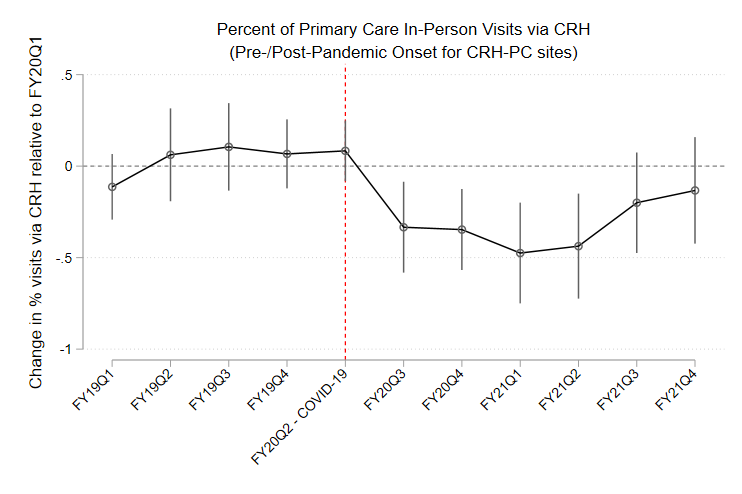 |
| VA primary care phone visits | VA primary care video visits |
| 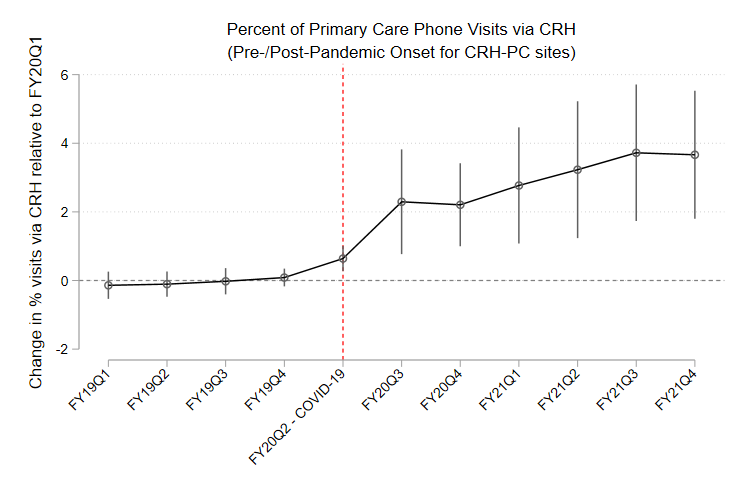 | 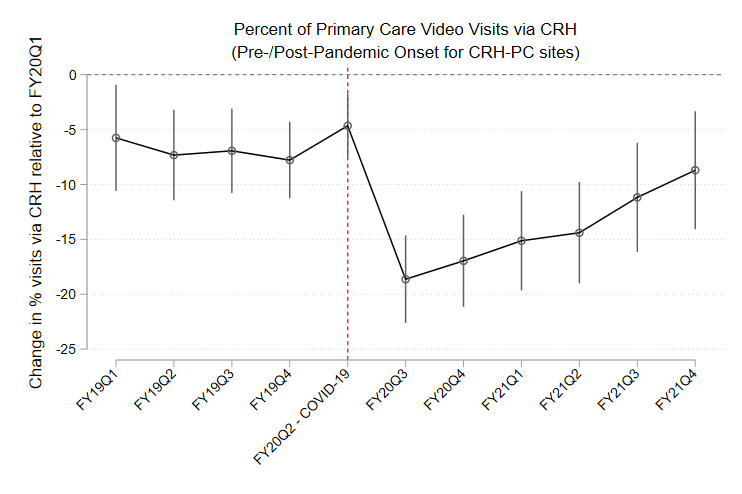 |
|  |  |

CRH PC sites, N = 254; Non-CRH PC sites, N = 0
